# Genital infections in high-risk human papillomavirus positive Paraguayan women aged 30–64 with and without cervical lesions

**DOI:** 10.1371/journal.pone.0312947

**Published:** 2024-10-29

**Authors:** Alanis Arévalos, Adriana Valenzuela, Pamela Mongelós, Hernán Barrios, María Isabel Rodríguez, Romina Báez, Claudia Centurión, Jaime Vester, Ana Soilán, Marina Ortega, Lorena Meza, Malvina Páez, Amalia Castro, Carmen Cristaldo, Ana Soskin, Gerardo Deluca, Armando Baena, Rolando Herrero, Maribel Almonte, Elena Kasamatsu, Laura Mendoza, ESTAMPA Paraguayan Study Group

**Affiliations:** 1 Instituto de Investigaciones en Ciencias de la Salud, Universidad Nacional de Asunción, San Lorenzo, Paraguay; 2 Hospital Nacional, Ministerio de Salud Pública y Bienestar Social, Itauguá, Paraguay; 3 Hospital General de San Lorenzo, Ministerio de Salud Pública y Bienestar Social, San Lorenzo, Paraguay; 4 Instituto Nacional del Cáncer, Ministerio de Salud Pública y Bienestar Social, Capiatá, Paraguay; 5 Instituto de Medicina Regional, Universidad Nacional del Noreste, Resistencia, Argentina; 6 International Agency for Research on Cancer, World Health Organization, Lyon, France; 7 Agencia Costarricense de Investigaciones Biomédicas (ACIB), Fundación INCIENSA, Nunciatura, Costa Rica; 8 Department of Noncommunicable Diseases, Rehabilitation and Disability, World Health Organization, Geneva, Switzerland; Universitat de Barcelona, SPAIN

## Abstract

**Objective:**

To determine the prevalence of genital infections (GIs), including sexual transmitted STIs: *Neisseria gonorrhoeae*, *Chlamydia trachomatis*, *Mycoplasma genitalium*, *Trichomonas vaginalis*, and opportunistic pathogens that generally do not cause STIs, non-classic STI: *Ureaplasma urealyticum*, *Ureaplasma parvum* and *Mycoplasma hominis*, in women with high-risk oncogenic human papillomavirus (hr-HPV) infection and their association with cervical lesions.

**Methods:**

A cross-sectional study was carried out including 231 hr-HPV positive women. Of these, 46 has histologically confirmed cervical intraepithelial neoplasia 3 (CIN3) or more (including CIN3 and cervical cancer lesions-CIN3+). GIs were detected by multiplex real time PCR. Odds ratios (OR) were estimated to explore possible associations between GIs and the presence or absence of CIN3+ lesions. Additionally, we examined associations between sociodemographic, sexual, and clinical characteristics and the presence of GIs.

**Results:**

In total, there were 174/231 cases of GIs corresponding to an overall prevalence of 75.3% (95%CI: 69.4–80.4), being non-classic STIs the most common (72.3%) compared to STIs (12.6%). The most prevalent non-classic STI and STI were *U*. *parvum* (49.8%) and *C*. *trachomatis* (7.4%), respectively. The odds of presenting GIs were 3 times higher in women under 46 years compared to older counterparts (OR: 3.32, 95%CI: 1.74–6.16), and in women with a normal Pap smear with inflammation compared to those without inflammation (OR: 3.31, 95%CI: 1.15–9.77). GIs were equally present in women with and without CIN3+ lesions.

**Conclusion:**

We observed an association of GIs with inflammation in the Pap smear, but no association with CIN3+, as some of them are very common and likely part of the normal vaginal flora, suggesting that such infections do not appear to be cofactors in cervical carcinogenesis, although larger prospective studies are needed.

## Introduction

Cervical cancer is the fourth most diagnosed type of cancer and the fourth most common cause of cancer death in women worldwide, with an estimated incidence of 660,000 new cases and 350,000 deaths in 2022, being the second cause of death from cancer in 45 countries, including Paraguay [1].

Cervical cancer is associated with persistent human papillomavirus (HPV) infections [2, 3]. HPV comprises a group of viruses that are extremely common worldwide, with more than 200 types. Based on HPV genotype-specific attributable fractions (AFs) in cervical cancer and their high prevalence in neoplastic lesions, 12 HPV types classified by IARC working group as Group 1 “carcinogenic HPV (cHPV) types” including HPV16, HPV18, HPV45, HPV33, HPV58, HPV31, HPV52, HPV35, HPV59, HPV39, HPV51 and HPV56, where HPV16 had the highest global AF in cervical cancer (61·7%), followed by HPV18 (15.3%), HPV45 (4.8%), HPV33 (3.8%), HPV58 (3.5%), HPV31 (2.8%), and HPV52 (2.8%), between others. In addition, HPV68 in alpha-7 is classified as probably carcinogenic to humans (Group 2A) and last, there are HPV types classified by IARC in Group 2B (possibly carcinogenic), some of which contribute very small attributable fractions and some of which cannot be attributed at all [2, 3].

Most screening-validated HPV tests detect between 13 to 14 HPV types including Group 2A and 2B HPV types such as 68 and 66, respectively, which are commonly known as hr-HPV types, with an approximate prevalence of 13.0% in Latin America. In the present study we use Hybrid Capture 2 HPV DNA Test (HC2) that detects 13 types (12 carcinogenic types plus HPV68) [4, 5].

HPV persistent infection is not sufficient for the development of cervical cancer; at least 80% of hr-HPV infections are transient and only a low percentage of women infected with hr-HPV are at risk of developing cervical intraepithelial neoplasia (CIN) and cervical cancer. There are cofactors that, along with HPV, can increase the risk of development of lesions and cervical cancer [6, 7]. Among these cofactors, we can find host-related elements such as hormonal and genetic background (that influence the ability of the immune system to eliminate the infection); virus-related factors such as genotype, viral load, coinfection of several HPV types and the integration of viral DNA into the host genome [7]. Additional cofactors for cervical cancer include history of smoking, younger age at first sexual intercourse and at first pregnancy, high parity, and long-term use of oral contraceptives [8–10].

Some authors claim that hr-HPV co-infection with *Chlamydia trachomatis*, *Ureaplasma urealyticum*, *Trichomonas vaginalis* or *Neisseria gonorrhoeae* increases the risk of developing abnormal cytology, pre-cancerous lesions and/or cervical cancer [11–14]. However, other authors have not observed a greater risk of developing cervical lesions among women co-infected by hr-HPV and other genital infections (GIs) [15–17]. Additionally, women could also have long-term health problems due to sexually transmitted infections (STIs) if left untreated. Chlamydia, gonorrhea, and trichomoniasis may cause infertility and adverse outcomes at delivery [18, 19].

Several *Mycoplasmataceae* including *Mycoplasma* and *Ureaplasma*, are relatively common GIs associated with cervical inflammation [20, 21]. Among *Mycoplasma*, only *M*. *genitalium* is sometimes included in regular STI screening, although *M*. *hominis* is believed to have a similar pathogenesis. As a result, *M*. *hominis* has received significantly less study, and its relationship with HPV, HIV and cervical lesions remains unclear. Klein *et al*. (2020) observed that *M*. *hominis* and *M*. *genitalium* infection were significantly more prevalent among women with HPV and HIV [21].

In the field of public health, it is very important to understand the role of co-factors (such as other GIs) on the development of cervical cancer in women infected with cHPV, in order to establish interventions for early detection and treatment of high-grade lesions and cancer. Considering that the detection of other GIs can be performed in the same sample collected for HPV test for cervical cancer screening in women 30 to 64 years-old, the objective of this study was to determine the prevalence of GIs in hr-HPV positive women with and without CIN3+ lesions (including: CIN3 or cervical cancer lesions) by multiplex real-time PCR, in order to provide data that could contribute with prevalence data of co-infections with hr-HPV and GIs, as well as, to better understand the role of GIs as co-factor to development pre-cancer lesions and cervical cancer.

## Materials and methods

This study presents partial results of the project: ¨Evaluation of molecular biomarkers for detection of the risk of persistent human papillomavirus-HPV infection and progression of cervical lesion in initially HPV-positive women with a diagnosis of CIN1 or less¨, that is part of the ESTAMPA study, conducted in Paraguay between 2014 and 2017 (ESTAMPA-PY) [22]. The ESTAMPA-Py study included 4120 women between 30 and 64 years of age screened using the HC2 test (QIAGEN, USA) and conventional cytology (Pap smear). The participants were selected from a census of the cities of San Lorenzo and Itauguá, Paraguay, and they had no history of cervical cancer, hysterectomy or prior treatment, as previously described in Kasamatsu *et al*., 2019 [23].

### Data and sample collection during screening

Sociodemographic characteristics, gynaecological and clinical history of the participating women were collected during screening. Two cervical brush samples were taken: one was used to prepare a conventional cytology (Pap smears), and the rest of the cells that remained on the brush were placed in a transport medium (ThinPrep PreservCyt Solution, HOLOGIC, USA) for the detection of hr-HPV by HC2 (QIAGEN, USA). The second brush was washed directly in the transport solution. All the volume was distributed in 8 cryovials and preserved at -80°C for other triage tests in ESTAMPA.

All 566/4120 women with positive screening results were invited to attend the colposcopy visit. 516 women (91.2%) attended the colposcopy visit. In the case of abnormal colposcopic impression, biopsy samples were taken and a diagnosis was made for each participant based on histology. The results were classified as: negative for squamous intraepithelial lesion (NSIL), cervical intraepithelial neoplasia (CIN) grade 1 (CIN 1, mild dysplasia), grade 2-CIN 2 (moderate dysplasia), grade 3-CIN 3 (severe dysplasia, carcinoma in situ) and invasive squamous carcinoma [22].

From these 516 women, 275 (53.3%) cervical samples had been used to evaluate other triage methods and did not have enough volume to perform the molecular GIs detection were not included in the present study. Furthermore, 10 cervical samples from women with CIN2 diagnosis without p16 results were excluded, considering that the diagnosis of CIN2 without p16 has little reproducibility, unlike the diagnosis of CIN3, to perform the analysis in the present study we only included CIN3 as pre-cancer cases [22].

### GIs detection

DNA from cervical samples was extracted using the “GeneJET Genomic DNA Purification Kit” (Thermo Scientific, USA), following the protocol indicated by the manufacturer. A multiplex real-time PCR was performed, using the “VIASURE Sexually Transmitted Diseases Real Time PCR Detection Kit” according to the protocol established by the manufacturer, for the detection of *N*. *gonorrhoeae*, *C*. *trachomatis*, *M*. *genitalium*, *T*. *vaginalis*, *U*. *urealyticum*, *U*. *parvum and M*. *hominis*. Each step of the process was carried out in a different room to avoid contamination.

### Interpretation of results

Tests return three potential results: positive, negative and invalid. Positive result: presence of amplification signal in any channel with Ct≤40. FAM channel: *C*. *trachomatis* and *T*. *vaginalis* (strip 1 or 2, respectively); ROX channel: *N*. *gonorrhoeae* and *U*. *parvum* (strip 1 or 2, respectively); HEX channel: *M*. *genitalium* and *U*. *urealyticum* (strip 1 or 2, respectively); Cy5 channel: *M*. *hominis* (strip 2). Negative result: absence of amplification signal in any channel. Invalid result: absence of signal in the internal control, presence of signal in the negative control and/or absence of signal in the positive control.

### Statistical analysis

Data analysis was conducted using descriptive statistics. The prevalence and 95% confidence intervals (95%CI) were calculated for each GI, including STIs (*C*. *trachomatis*, *T*. *vaginalis*, *N*. *gonorrhoeae*, and *M*. *genitalium*), and non-classic STIs, which include opportunistic pathogens not typically associated with STIs (*U*. *urealyticum*, *U*. *parvum*, and *M*. *hominis*). Prevalence was determined for all GIs, as well as for co-infections (two or more GIs), in both groups: hr-HPV positive women with and without CIN3+ lesions. Additionally, we examined associations between sociodemographic, sexual, and clinical characteristics and the presence of GIs in hr-HPV positive women, using odds ratios (OR) obtained from contingency tables. For these analyses, GIs were also categorized by STIs and non-classic STIs. Furthermore, the presence of any GI (regardless of whether it was a STI or non-classic STI) was analyzed in relation to the presence or absence of CIN3+ lesions. Both crude ORs with 95%CI and p-values (two-sided) from Fisher’s Exact test were calculated for categorical variables, and the Mann-Whitney test was used for numerical variables to assess statistical significance. Statistical analysis was performed using GraphPad Prism version 10.1.2.

### Ethical issues

The project was approval by the Scientific Committee and Ethics Committee of the IICS—UNA, with code P09/2018. The women participating in the ESTAMPA-Py study have signed an informed consent prior to sample collection, which detailed the procedures and risks and benefits of their participation, and where they expressed their agreement that their samples be stored using codes and that they can be used in the future in the evaluation of new strategies that can serve to improve the early detection of cervical cancer. The identities of the women were kept confidential, using a numerical code for identification of samples and for all sociodemographic, gynaecological and clinical data´s collected of the ESTAMPA-Py study participating women. Therefore, for this study, the researchers had access to the data´s collected under codes after obtaining the approval of the Scientific and Ethics Committees of the project mentioned above (code P9/2018) on date 22/06/2020.

## Results

**Fig 1:**
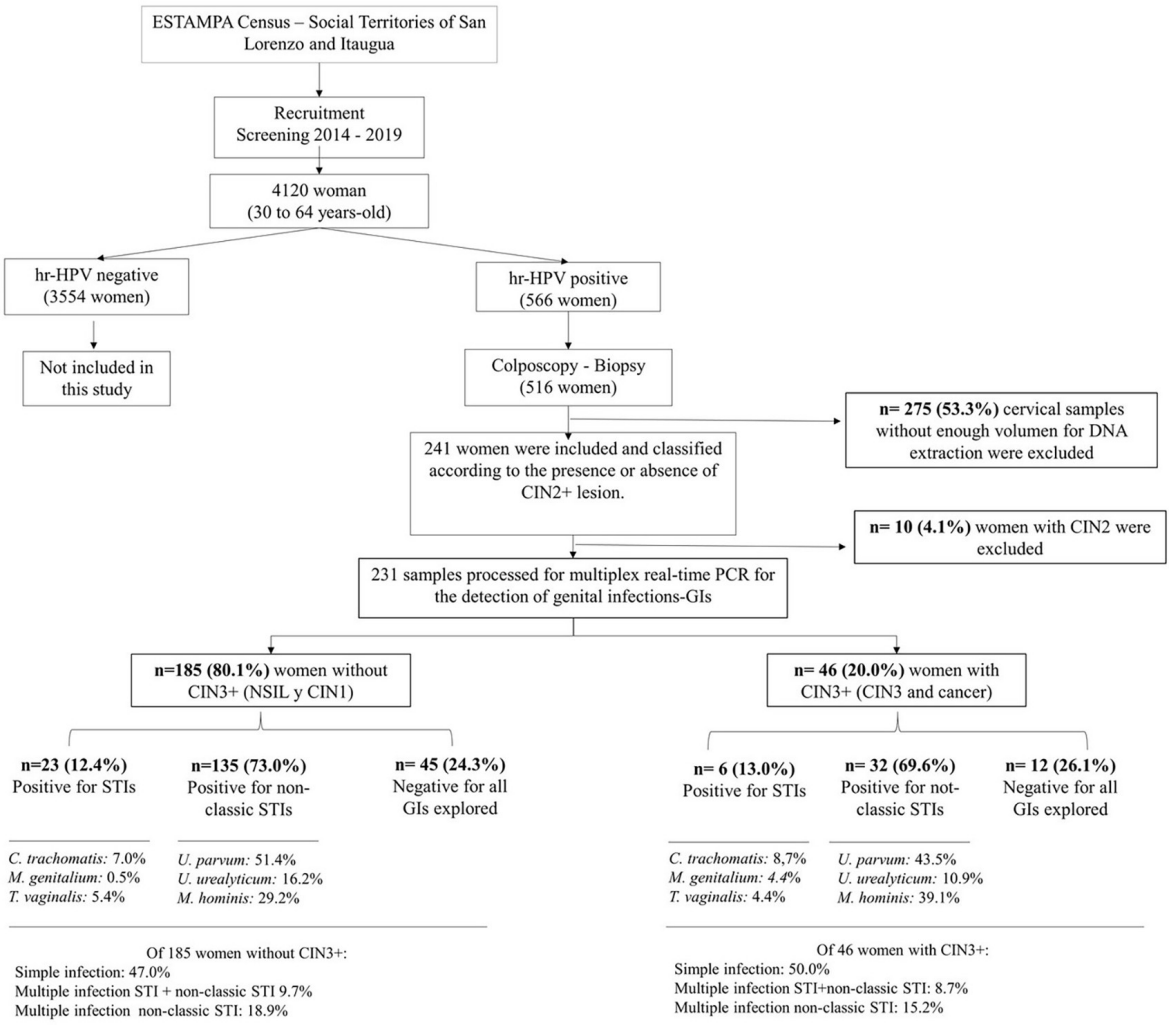
Flowchart of the participant selection process.

The analysis is based on results from 231 hr-HPV positive women included in this study: 151 women with a diagnosis of NSIL, 34 women with CIN1, 40 women with CIN3 and 6 women with cervical cancer. They were classified into two groups: 46 women in the group “with CIN3+ lesion” (those with a diagnosis of CIN3 and cervical cancer), and 185 women in the group “without CIN3+ lesions” (those with a diagnosis of NSIL or CIN1). The flowchart of the participant selection process is presented in Fig 1.

The sociodemographic, sexual and clinical characteristics are described in Table 1. The median age of participants was 40 years. The majority (64.5%) reported that they had never attended school or that they had not completed it. Additionally, 67.5% of participants reported having 2 or more sexual partners. Furthermore, 72.3% of all women included presented normal screening Pap smears with a high percentage (90.4%) of inflammation reported.

**Table 1:** Sociodemographic, sexual and clinical characteristics of 231 women with hr-HPV infection.

Characteristic	hr-HPV positive women (n = 231)	
	N	% (95% CI)
Age (years)		
Median (IQR25%-75%)	231	40.0 (34.0–48.0)
**First sexual intercourse**		
Median (IQR25%-75%)	231	18.0 (16.0–20.0)
<18 years	115	49.4 (43.1–55.6)
≥18 years	116	50.6 (44.3–56.9)
**Education**		
Not received/Incomplete	149	63.1 (56.8–68.9)
Complete secondary school or more	81	36.5 (30.7–42.8)
Missing data	1	0.4 (0.2–2.3)
**Previous Pap smear**		
Yes	221	95.9 (92.5–97.7)
No	10	4.1 (2.3–7.5)
**Pap screening smear**		
Normal	167	71.8 (65.8–77.1)
Abnormal-ASCUS+^1^	62	27.4 (22.1–33.3)
Unsatisfactory quality	2	0.8 (0.1–3.0)
**Normal Pap screening smear**		
Normal without inflammation	16	9.8 (6.2–15.2)
Normal with inflammation	151	90.2 (84.8–93.8)
**Smoking habit**		
Yes	36	15.8 (11.7–20.9)
No	195	84.2 (79.1–88.3)
**Condom use**		
Yes	87	39.0 (33.1–45.3)
No	144	61.0 (54.7–66.9)
**n sexual partners**		
Median (IQR25%-75%)	215	2.0 (1.0–4.0)
1	59	25.3 (20.0–31.2)
≥2	156	68.0 (61.9–73.6)
Missing data	16	6.6 (4.1–10.5)
**n pregnancies**		
Median (IQR25%-75%)	231	3.0 (2.0–5.0)
<3	71	32.9 (27.2–38.9)
≥3	160	67.2 (61.1–72.8)
**n women with/without CIN3+ lesions** ^**2**^		
Women without CIN3+ lesión	185	76.8 (71.0–81.6)
Women with CIN3+	46	19.1 (14.6–24.5)

hr-HPV: high-risk human papillomavirus. CIN: cervical intraepitelial neoplasia. 231 hr-HPV positive women including: 151 women negative for CIN, 34 with CIN1, 40 with CIN3 and 6 with cervical cancer.

^1^62 women with abnormal cytology-ASCUS+, including: 11 Atypical Squamous Cells of Undetermined Significance (ASC-US); 8 Atypical Squamous Cells, cannot rule out high grade squamous intra-epithelial lesion (ASC-H); 26 low-grade squamous intraepithelial lesion (LSIL); 17 high-grade squamous intraepithelial lesion (HSIL).

^2^Women without CIN3+ lesion: 151 women negative for CIN lesion and 34 with CIN1 lesion. Women with CIN3+ lesion: 40 women with CIN3 lesion and 6 with cervical cancer.

IQR 25%-75%: interquartile range 25% - 75%. 95%CI: 95% confidence interval.

In total, 174 women had GIs, corresponding to an overall prevalence of 75.3% (95%CI: 69.4–80.4). Non-classic STIs were the most common GIs (72.3%) compared to STIs (12.6%). The detection of specific GIs in 231 women is shown in Fig 1. The most prevalent STI was *C*. *trachomatis* (7.4%), followed by *T*. *vaginalis* (5.2%). Among non-classic STIs, *U*. *parvum* was the most prevalent (49.8%), followed by *M*. *hominis* (31.2%). *N*. *gonorrhoeae* was not detected in any case. Additionally, co-infections with two or more GIs were found in 37.4% of hr-HPV positive women.

We also described the sociodemographic, sexual, and clinical characteristics of hr-HPV-positive women with and without GIs; results are presented in Table 2. The odds of presenting GIs were significantly 3-fold higher in women under 46 years compared to older counterparts (OR: 3.32 (95%CI: 1.74–6.16), and in women with a normal Pap smear showing inflammatory reactive changes compared to those without inflammation (OR: 3.31, 95%CI: 1.15–9.77). The presence of GIs did not significantly differ according to other participants’ characteristics.

**Table 2:** Sociodemographic, sexual and clinical characteristics in hr-HPV positive women with and without other genital infections.

Characteristic	hr-HPV with STI (n = 29)		hr-HPV without another GI (n = 57)					hr-HPV with non-classic STI (n = 167)		hr-HPV without another GI (n = 57)				hr-HPV with GI (n = 174)		hr-HPV without another GI (n = 57)			
	N	% (95% CI)	N	% (95% CI)	P	OR	N	% (95% CI)	n	% (95% CI)	p	OR	n	% (95% CI)	n	% (95% CI)	p	OR
**Prevalence n total = 231**	29	12.6 (8.9–17.4)	57	24.7 (19.6–30.6)			167	72.3 (66.2–77.7)	57	24.7 (19.6–30.6)			174	75.3 (69.4–80.4)	57	24.7 (19.6–30.6)		
**Age (years)**																		
Median (IQR25%-75%)	29	34.0 (32.0–46.5)	57	46.0 (36.5–51.0)	0.0133*		167	39.0 (34.0–47.0)	57	46.0 (36.5–51.0)	0.0069*		174	39.0 (34.0–47.0)	57	46.0 (36.5–51.0)	0.0091*	
46–64 years	7	24.1 (12.2–42.1)	31	54.4 (41.6–66.6)	0.0111*	1	43	25.7 (19.7–32.9)	31	54.4 (41.6–66.6)	0.0001*	1	46	26.4 (20.4–33.4)	31	54.4 (41.6–66.6)	0.0002*	1
30–45 years	22	75.9 (57.9–87.8)	26	45.6 (33.4–58.4)		3.75 (1.32–9.34)	124	74.2 (67.1–80.3)	26	45.6 (33.4–58.4)		3.44 (1.87–6.46)	128	73.6 (66.6–79.6)	26	45.6 (33.4–58.4)		3.32 (1.74–6.16)
**First sexual intercourse**																		
≥18 years	12	41.4 (25.5–59.3)	35	61.4 (48.4–72.9)	0.11	1	77	46.1 (38.7–53.7)	35	61.4 (48.4–72.9)	0.0651	1	81	46.5 (39.3–54.0)	35	61.4 (48.4–72.9)	0.0667	1
<18 years	17	58.6 (40.7–74.5)	22	38.6 (27.1–51.6)		2.25 (0.87–5.62)	90	53.9 (46.3–61.3)	22	38.6 (27.1–51.6)		1.86 (1.00–3.46)	93	53.4 (46,0–60.7)	22	38.6 (27.1–51.6)		1.83 (0.99–3.38)
**Education**																		
Complete secondary school or more	11	37.9 (22.7–56.0)	22	38.6 (27.1–51.6)	>0.999	1	55	32.9 (26.3–40.4)	22	38.6 (27.1–51.6)	0.42	1	59	33.9 (27.3–41.2)	22	38.6 (27.1–51.6)	0.52	1
Not received/Incomplete	18	62.1 (44.0–77.3)	34	59.6 (46.7–71.4)		1.06 (0.44–2.73)	112	67.1 (59.6–73.7)	34	59.6 (46.7–71.4)		1.32 (0.69–2.40)	115	66.1 (58.8–72.7)	34	59.6 (46.7–71.4)		1.26 (0.67–2.39)
No data	0	0.0 (0.0–11.7)	1	1.8 (0.1–9.3)			0	0.0 (0.0–2.2)	1	1.8 (0.1–9.3)			0	0.0 (0.0–2.2)	1	1.8 (0.1–9.3)		
**Previous cytology**																		
No	3	10.3 (3.6–26.4)	0	0.0 (0.0–6.3)	0.0357*	1	9	5.4 (2.9–9.9)	0	0.0 (0.0–6.3)	0.12	1	10	5.7 (3.2–10.3)	0	0.0 (0.0–6.3)	0.12	1
Yes	26	89.7 (73.6–96.4)	57	100.0 (93.7–100.0)		0.00 (0.00–0.56)	158	94.6 (90.1–97.1)	57	100.0 (93.7–100.0)		0.00 (0.00–1.11)	164	94.3 (89.7–96.8)	57	100.0 (93.7–100.0)		0.00 (0.00–1.00)
**Pap screening result**																		
Abnormal-ASCUS +	1	3.4 (0.2–17.2)	14	24.6 (15.2–37.1)	0.0298*	1	48	28.7 (22.4–36.0)	14	24.6 (15.2–37.1)	0.61	1	48	27.6 (21.5–34.7)	14	24.6 (15.2–37.1)	0.73	1
Normal	26	89.7 (73.6–96.4)	43	75.4 (62.9–84.8)		8.47 (1.38–92.94)	118	70.7 (63.3–77.0)	43	75.4 (62.9–84.8)		0.80 (0.40–1.59)	124	71.3 (64.1–77.5)	43	75.4 (62.9–84.8)		1.19 (0.60–2.40)
Unsatisfactory quality	2	6.9 (1.2–22.0)	0	0.0 (0.0–6.3)			1	0.6 (0.0–3.3)	0	0.0 (0.0–6.3)			2	1.1 (0.2–4.1)	0	0.0 (0.0–6.3)		
**Normal Pap screening result**																		
Normal without reactive changes	1	3.8 (0.2–18.9)	8	18.6 (9.7–32.6)	0.14	1	8	6.8 (3.5–12.8)	8	18.6 (9.7–32.6)	0.0369*	1	8	7.0 (3.7–12.8)	8	17.8 (9.3–31.3)	0.0320*	1
Normal with reactive changes -exhibiting inflamed cells	25	92.2 (81.1–99.8)	35	81.4 (67.4–90.3)		5.71 (0.91–65.67)	110	93.2 (87.2–96.5)	35	81.4 (67.4–90.3)		3.14 (1.06–9.28)	116	93.0 (87.2–96.3)	35	82.2 (68.7–90.7)		3.31 (1.15–9.77)
**Smoking habit**																		
No	24	82.8 (65.4–92.4)	48	84.2 (72.6–91.5)	>0.999	1	141	84.4 (78.2–89.1)	48	84.2 (72.6–91.5)	>0,999	1	147	84.5 (78.4–89.1)	48	84.2 (72.6–91.5)	>0.999	1
Yes	5	17.2 (7.6–34.5)	9	15.8 (8.5–27.4)		1.11 (0.37–3.87)	26	15.6 (10.8–21.8)	9	15.8 (8.5–27.4)		0.98 (0.44–2.34)	27	15.5 (10.9–21.6)	9	15.8 (8.5–27.4)		0.98 (0.45–2.32)
**Condom use**																		
Yes	8	27.6 (14.7–45.7)	22	38.6 (27.0–51.6)	0.35	1	62	37.1 (30.2–44.7)	22	38.6 (27.0–51.6)	0.87	1	65	37.4 (30.5–44.7)	22	38.6 (27.0–51.6)	0.88	1
No	21	72.4 (54.3–85.3)	35	61.4 (48.4–72.9)		1.65 (0.66–4.45)	105	62.9 (55.3–69.8)	35	61.4 (48.4–72.9)		1.06 (0.57–2.00)	109	62.6 (55.3–69.5)	35	61.4 (48.4–72.9)		0.95 (0.51–1.77)
**n sexual partners**																		
≥2	18	62.1 (44.0–77.3)	39	68.4 (55.5–80.0)	0.62	1	115	68.9 (61.5–75.4)	39	68.4 (55.5–80.0)	0.60	1	117	67.2 (60.0–73.8)	39	68.4 (55.5–80.0)	0.73	1
1	10	34.5 (19.9–52.7)	16	28.1 (18.1–40.8)		1.35 (0.54–3.54)	39	23.3 (17.6–30.3)	16	28.1 (18.1–40.8)		0.83 (0.41–1.60)	43	24.7 (18.9–31.6)	16	28.1 (18.1–40.8)		0.90 (0.45–1.72)
Missing data	1	3.4 (0.2–17.2)	2	3.5 (0.6–11.9)			13	7.8 (4.6–12.9)	2	3.5 (0.6–11.9)			14	8.0 (4.8–13.0)	2	3.5 (0.6–11.9)		
**Pregnancies**																		
≥3	20	69.0 (50.8–82.7)	43	75.4 (62.9–84.8)	0.61	1	112	67.1 (59.6–73.7)	43	75.4 (62.9–84.8)	0.25	1	117	67.2 (60.0–73.8)	43	75.4 (62.9–84.8)	0.32	1
<3	9	31.0 (17.3–49.2)	14	24.6 (15.2–37.1)		1.38 (0.53–3.77)	55	32.9 (26.3–40.4)	14	24.6 (15.2–37.1)		1.51 (0.77–3.01)	57	32.8 (26.2–40.0)	14	24.6 (15.2–37.1)		1.50 (0.77–2.97)

hr-HPV: high-risk human papillomavirus. STI: sexually transmitted infection (*C*. *trachomatis*, *T*. *vaginalis* and/or *M*. *genitalium*); non-classic STI: *M*. *hominis*. *U*. *urealyticum* and/or *U*. *parvum*; GIs: all genital infections analyzed in this study.

IQR25%-75%: interquartile range 25% - 75%. 95%CI: 95% confidence interval.

*p<0.05 (statistically significant). OR: Odds ratio.

The presence of GIs observed in women with and without CIN3+ lesion is shown in Table 3. GIs were equally present in women with and without CIN3+ lesions.

**Table 3:** Prevalence of infections in hr-HPV positive women with and without CIN3+.

Genital infections	hr-HPV positive women (n = 231)		hr-HPV without CIN3+ lesion (n = 185)		hr-HPV with CIN3+ lesion (n = 46)			
	N	% (95% CI)	n	% (95% CI)	N	% (95% CI)	P	OR
**Sexually transmited infections-STI**								
***N*. *gonorrhoeae***								
Negative	231	100.0 (98.4–100.0)	185	100.0 (98.0–100.0)	46	100.0 (92.3–100.0)	-	-
Positive	0	0.0 (0.0–1.6)	0	0.0 (0.0–2.0)	0	0.0 (0.0–7.7)		
***C*. *trachomatis***								
Negative	214	92.6 (88.5–95.3)	172	93.0 (88.3–95.8)	42	91.3 (79.7–96.6)	0.75	1
Positive	17	7.4 (4.6–11.5)	13	7.0 (4.2–11.7)	4	8.7 (3.4–20.3)		0.79 (0.24–2.32)
***M*. *genitalium***								
Negative	228	98.7 (96.2–99.6)	184	99.5 (97.0–100.0)	44	95.6 (85.5–99.2)	0.10	1
Positive	3	1.3 (0.3–3.7)	1	0.5 (0.0–3.0)	2	4.4 (0.8–14.5)		0.12 (0.01–1.06)
***T*. *vaginalis***								
Negative	219	94.8 (91.1–97.0)	175	94.6 (90.3–97.0)	44	95.6 (85.5–99.2)	>0.999	1
Positive	12	5.2 (3.0–8.9)	10	5.4 (3.0–9.7)	2	4.4 (0.8–14.5)		1.26 (0.30–5.90)
**Non-classic STI**								
***U*. *parvum***								
Negative	116	50.2 (43.8–56.6)	90	48.6 (41.5–55.8)	26	56.5 (42.2–69.8)	0.41	1
Positive	115	49.8 (43.4–56.2)	95	51.4 (44.2–58.4)	20	43.5 (30.2–57.7)		1.37 (0.71–2.58)
***U*. *urealyticum***								
Negative	196	84.8 (79.7–88.9)	155	83.8 (77.8–88.4)	41	89.1 (77.0–95.3)	0.49	1
Positive	35	15.2 (11.1–20.3)	30	16.2 (11.6–22.2)	5	10.9 (4.7–23.0)		1.59 (0.62–3.96)
***M*. *hominis***								
Negative	159	68.8 (62.6–74.5)	131	70.8 (63.9–76.9)	28	60.9 (46.5–73.6)	0.21	1
Positive	72	31.2 (25.5–37.4)	54	29.2 (23.1–36.1)	18	39.1 (26.4–53.5)		0.64 (0.32–1.28)
**GI**								
Negative for all GI	57	24.7 (19.6–30.6)	45	24.3 (18.7–31.0)	12	26.1 (15.6–40.3)	0.85	1
Positive for at least one GI	174	75.3 (69.4–80.4)	140	75.7 (69.0–81.3)	34	73.9 (59.7–84.4)		1.10 (0.53–2.25)
**Coinfections**								
Coinfection with one GI	109	62.6 (55.3–69.5)	87	62.1 (53.9–69.8)	22	64.7 (47.9–78.5)	0,84	1
Coinfection with 2 or more GIs	65	37.4 (30.5–44.7)	53	37.9 (30.2–46.1)	12	35.3 (21.5–52.1)		1.12 (0.52–2.49)

hr-HPV: high-risk human papillomavirus. STI: sexually transmitted infection: *C*. *trachomatis*, *T*. *vaginalis* and/or *M*. *genitalium*. non-classic STI: *M*. *hominis*. *U*. *urealyticum* and/or *U*. *parvum*. GI: all genital infections analyzed. CIN: cervical intraepitelial neoplasia. hr-HPV positive women: 151 women negative for CIN, 34 with CIN1, 40 with CIN3 and 6 with cervical cancer. hr-HPV positive women without CIN3+ lesion:151 women negative for CIN lesion and 34 with CIN1 lesion. hr-HPV positive women with CIN3+ lesion: 40 women with CIN3 lesion and 6 with cervical cancer. 95%CI: 95% confidence interval. OR: Odds ratio.

## Discussion

There are few studies on the prevalence of other GIs in hr-HPV positive women and their role in the development of cervical neoplasia. Therefore, the objective of the present study was to determine the prevalence of GIs in hr-HPV positive women and their association with cervical lesions.

Studies have shown that HPV positive women had a slightly higher GIs rate than the HPV-negative group [24–26], which, although we did not test HPV negative women, could partly explain the high percentage of normal Pap smears with inflammation (93.0%) observed in hr-HPV positive women in the present study, as well as the high prevalence of other GIs (75.3%) detected, even higher than reported in other populations in Latin America. In indigenous women from Paraguay, 51.7% of those infected with hr-HPV had bacterial vaginosis, which is associated with infections by *C*. *trachomatis*, *T*. *vaginalis* and *M*. *hominis*; while in a study carried out in rural areas of Brazil, 43.3% of HPV positive women had another GIs [27, 28]. These differences could be due to discrepancies in the number of GIs investigated: in the work carried out in Brazil, only 4 GIs were detected. In this study, we used real time PCR which has higher sensitivity (93% to 100%) to detect GIs compared to conventional methods of culture and microscopy. In addition, they can be due to differences in sexual behaviour and the use of preventive methods such as condoms between different populations.

GIs, including HPV infection, are transmitted mainly sexually, thus the risk of acquiring the infection decreases with age, and there is a higher probability of eliminating the infection over time. The prevalence of HPV infection reaches a peak in those under 30 years of age [29]. This is also observed in the case of other GIs: Kim *et al*. (2016) studied the same 7 GIs that were analyzed in the present study, and they observed that the prevalence of infection by all GIs together, as well as by *C*. *trachomatis*, *M*. *genitalium* and *U*. *parvum* were higher in women under 50 years of age [30]. In this study, the prevalence of GIs was higher in women younger than 46 years vs older counterparts (OR: 3.32).

The study by Remschmidt *et al*. (2014) observed that factors strongly associated with younger age at first sexual intercourse were: a high number (≥ 7) of sexual partners during lifetime, current smoking and a past pregnancy, concluding that younger age at first sexual intercourse was associated with behavior that might increase the risk of HPV infections or other STIs [31]. In the present study, we did not detect an association between the number of sexual partners and a higher prevalence of other GIs in hr-HPV positive women; this could be due to the impossibility of stratifying the number of sexual partners into more groups due to the sample size and the fact that all women are HPV positive.

The prevalence of GIs was significantly higher in women with normal Pap smear with inflammation vs without inflammation (OR: 3.31). Studies have shown that a more diverse vaginal microbiota with a high amount of non-*Lactobacillus spp*. (dysbiosis) has been associated with local inflammation, characterized by an increase in pro-inflammatory cytokines and the presence of activated cells, which in turn increase the susceptibility to STIs and poor obstetrics event that could explain in part the association observed between inflammation and GIs in the present study [32, 33].

Other studies observed association between GIs and Pap smears with inflammation [34–36]. The Pap smears remains the main screening method in Paraguay. However, Paraguay is currently expanding access to HPV tests as primary screening, and in these regions cytology can be used as a triage test. The high prevalence of others GIs in hr-HPV positive women (75.3%) observed in the present study suggest the relevance of strengthening the detection of GIs, especially in women with normal Pap smears with inflammation, to increase the treatment of necessary cases in time and avoid possible complications.

A key limitation of this study is the small sample size in certain subgroups, such as the STIs group (n = 29). This may lead to the underrepresentation of some explanatory variable categories and outcomes, partially explaining the wide 95%CIs for ORs, such as those for previous Pap tests and screening results, where only three and one women with STIs were in the reference groups (no previous cytology and abnormal ASCUS+, respectively). This highlights the need for further studies with larger sample sizes to obtain more reliable results.

Furthermore, there were no significant differences between the prevalence of GIs detected in hr-HPV positive women with and without CIN3+ lesion, and with those obtained in studies conducted in Spain and Colombia that reported that, among HPV-positive women, positive serology for other GIs (including *C*. *trachomatis and N*. *gonorrhoeae)* did not increase the risk of developing CIN3 or invasive cancer [16]. Castle *et al*. (2003) found no association between HPV and *C*. *trachomatis* co-infection and high grade lesions [15]. From 50 to 80% of sexually active individuals become infected with both *C*. *trachomatis* and HPV during their lifetime, and up to 50% become infected with an hr-HPV [37]. Data have revealed up to a four-fold higher risk of hr-HPV infection in *C*. *trachomatis*-positive women compared to the negative group, and nearly a two-fold duration of the hr-HPV infection [38]. The co-infection with *C*. *trachomatis* and HPV (especially the 16, 18, 31, 33, 53, and 56 genotypes) is considered by some studies a risk factor for cervical cancer [39–43].

However, others epidemiological studies, have reported no association [15, 16, 44, 45]. Even though both HPV and *C*. *trachomatis* share common transmission routes and risk factors, there is a lack of physiologically relevant infection models that could clarify the mechanisms of the infection’s progression and the development of cancer. Furthermore, there is a need to investigate additional factors that could eventually be responsible for the process, such as the role of local microbiota and the immunity status [37, 38].

There are studies that report that women who have co-infection with hr-HPV and another GIs have a higher risk of developing high grade lesions and cervical cancer [46]. The discrepancy in the results may be explained by differences in the clinical characteristics of the women included. In addition, when comparing between two groups of women infected with hr-HPV, it is possible that the group “without CIN3+ lesion” included women that have recently acquired the infection, so there was not enough time yet for the development of lesions, which could have affected the results. The existing controversy demonstrates the need to perform long-term analytical and follow-up studies in order to clarify the role of GIs as risk cofactor in the development of CIN3+ lesions.

The most frequently detected GIs in the study were non-classic STIs of the genus *Mycoplasma* and *Ureaplasma*, that are relatively common in genital area and they are generally associated with cervical inflammation, which could partly explain why hr-HPV positive women with and without CIN3+ have a similar prevalence of these infections [19, 20]. This is in accordance with the study by Klein *et al*. (2020) that observed that *M*. *hominis* prevalence was similar despite severity of cervical lesions, however *M*. *hominis* proportion increased significantly in women with cervical lesions [21]. These results emphasize the importance of conducting more studies to better understand the role of these infections as a risk factor for the development of CIN3+ lesions.

Finally, despite the limited sample size, especially in the STIs group, and the exclusion of several samples due to insufficient volume, our results align with larger studies. This suggests that the controversy in the literature regarding the association between STIs and cervical pre-cancer or cancer may be influenced by other factors, highlighting the need for larger prospective multicentric studies [15, 16, 44, 45].

In conclusion, the increased prevalence of GIs explored in hr-HPV-positive women with normal Pap smears with inflammation, and the lack of association with high-grade cervical lesions and cancer, suggest that such infections do not appear to be cofactors in cervical carcinogenesis, although larger prospective studies are needed.
